# Cortisol, Anxiety, and TNFα Mediate the Relationship Between BMI and Executive Functions

**DOI:** 10.1002/smi.70077

**Published:** 2025-07-12

**Authors:** Anna Prunell‐Castañé, María Ángeles Jurado, Xavier Caldú, María José Sender‐Palacios, Consuelo Sánchez Garre, Paloma Salas Gómez‐Pablos, Maite Garolera

**Affiliations:** ^1^ Departament de Psicologia Clínica i Psicobiologia Facultat de Psicologia Universitat de Barcelona Passeig de la Vall D’Hebron Barcelona Spain; ^2^ Institut de Neurociències Universitat de Barcelona Barcelona Spain; ^3^ Institut de Recerca Sant Joan de Déu Esplugues de Llobregat Barcelona Spain; ^4^ Primary Care Health Center Terrassa Nord Consorci Sanitari de Terrassa Terrassa Spain; ^5^ Pediatric Endocrinology Unit Hospital de Terrassa Consorci Sanitari de Terrassa Terrassa Spain; ^6^ Catlab Consorci Sanitari de Terrassa Terrassa Spain; ^7^ Brain Cognition and Behavior: Clinical Research Hospital de Terrassa Consorci Sanitari de Terrassa Terrassa Spain; ^8^ Neuropsychology Unit Hospital de Terrassa Consorci Sanitari de Terrassa Terrassa Spain

**Keywords:** anxiety, body mass index, cortisol, executive functions, TNFα

## Abstract

Overweight and obesity are associated with poorer executive functions (EF). The underlying mechanisms contributing to this relationship are not yet conclusive, but cortisol, anxiety, and inflammation are likely among the contributing factors. Our objective was to evaluate whether fibrinogen, tumour necrosis factor alpha (TNFα), cortisol, and anxiety significantly mediate the association between body mass index (BMI) z‐score and EF (i.e., working memory, inhibition, cognitive flexibility, and a latent EF component) in both adolescents and adults. In this cross‐sectional study, 268 participants aged 11–49 years with BMI values ranging from normal‐weight to obesity were medically and neuropsychologically evaluated. Moderated multiple mediation analyses with mediators in parallel were conducted (X: BMI z‐score; M: cortisol, anxiety, TNFα, and fibrinogen; Y: executive functions; Moderator: adolescent and adult groups). Our results suggested that TNFα mediated the association between BMI z‐score and working memory only in adolescents (indirect effect = 0.09, 95% CI [0.03, 0.19]), whereas in adults, cortisol (indirect effect = −0.06, 95% CI [−0.13, −0.01]) and anxiety (indirect effect = 0.02, 95% CI [0.0001, 0.07]) mediated this association. Additionally, in adults, anxiety also mediated the relationship between BMI z‐score and the latent EF component (indirect effect = 0.03, 95% CI [0.004, 0.08]). In the full sample, TNFα significantly mediated the relationship between BMI z‐score and inhibition (indirect effect = −0.03, 95% CI [−0.06, −0.002]). In conclusion, our study suggests that inflammation, cortisol, and anxiety are biologically and psychologically plausible mechanisms through which BMI may influence cognitive performance. Large‐scale longitudinal studies are needed to confirm these findings and to determine whether the observed associations are age‐dependent.

## 1 Introduction

The prevalence of overweight and obesity is increasing at an alarming rate, affecting more than one third of the world's population (Chooi et al. 2019). Overweight and obesity lead to multimorbidity (Kivimäki et al. 2022), together with cognitive decline (Jung and Mok 2022). Core executive functions (EF), namely working memory, inhibition, and cognitive flexibility, encompass cognitive processes that facilitate the achievement of goals (Diamond 2013), including the self‐regulation of eating behaviour (e.g., redirecting attention away from tempting stimuli, limiting impulsive responses, and choosing the most adaptative method to maintain healthy eating) (Dohle et al. 2018). Higher body mass index (BMI) categories have consistently been associated with poorer EF (Favieri et al. 2019). Understanding the underlying mechanisms of this relationship is crucial, as it may inform obesity prevention and management strategies. Although the current literature is not yet conclusive, inflammation, cortisol, and anxiety are likely among the contributing factors.

Obesity has been associated with both adipose tissue inflammation and systemic low‐grade inflammation, which are associated with a persistent immune system activation. What triggers the latter, according to the prevailing hypothesis, is the presence of lipopolysaccharides (LPS) in the bloodstream, a condition known as endotoxaemia (Soták et al. 2025). Two possible sources of such microbial products have been described: first, periodontitis, which is broadly associated with cardiovascular disease; and second, intestine dysbiosis, which may compromise the gut barrier and allow LPS to enter the circulation (Soták et al. 2025). Additionally, ectopic lipid deposition in the liver and physical inactivity have also been described as promoters of systemic low‐grade inflammation. In contrast, obesity‐related adipose tissue inflammation is primarily driven by its expansion, which involves processes such as adipocyte hypertrophy, hypoxia, and necrosis, and is exacerbated by systemic low‐grade inflammation and endotoxaemia (Soták et al. 2025). In both cases, the resulting inflammatory state is characterised by the presence of proinflammatory cytokines, such as tumour necrosis factor alpha (TNFα), and other pro‐inflammatory agents, like C‐reactive protein (CRP) and fibrinogen (Ellulu et al. 2017).

How inflammation can impact EF was approached by a recent theoretical model, the immunologic model of self‐regulatory failure. This model proposed that inflammatory activity, specially proinflammatory cytokines, can directly influence neuronal activity by binding to cytokine receptors on neurons, or indirectly affect brain by stimulation of the vagus nerve or by disrupting the synthesis and breakdown of various neurotransmitters (Shields et al. 2017).

The relationship between inflammatory activity and brain structure in regions involved in EF, such as the dorsolateral prefrontal cortex (Aydin et al. 2024) and the anterior cingulate cortex (van Velzen et al. 2017), has already been documented. Functional alterations in the central executive network (Nusslock et al. 2019), as well as in connectivity between the right inferior frontal gyrus and the left parietal cortex (Swartz et al. 2021) have also been reported in the context of inflammation. Recent evidence has also described the mediating role of inflammatory biomarkers on the relationship between obesity and EF (Cannavale et al. 2021; King et al. 2023; Shields et al. 2021; Yang et al. 2020).

Obesity has also been commonly associated with a dysregulated hypothalamic‐pituitary‐adrenal axis (Incollingo Rodriguez et al. 2015). Cortisol, its end product, can bind to glucocorticoid (GR) and mineralocorticoid (MR) receptors in target tissues, affecting physiological processes such as glucose and lipid metabolism, immune function, and stress responses (Lengton et al. 2024). Due to its lipophilic structure, cortisol can also cross the blood‐brain barrier and enter the central nervous system. The brain is rich in both MR and GR; however, within the prefrontal cortex—the region primarily responsible for EF—only GR are expressed. The effects of these two receptor types on cognitive function are generally opposite: MR are associated with positive effects, while GR have been linked to negative outcomes. Consequently, elevated cortisol levels may impair EF (Ouanes and Popp 2019), although mixed results have also been reported (Shields et al. 2015). A recent study found correlational evidence between acute overnight hydrocortisone administration and decreased perfusion in brain regions central to EF, including the anterior cingulate cortex, dorsolateral prefrontal cortex, orbitofrontal cortex, and ventromedial prefrontal cortex (Bini et al. 2022). Similarly, another study reported that higher cortisol levels were linked to reduced grey matter volume in the frontal lobe and other regions in adults with a wide range of BMI, from normal‐weight to obesity (Echouffo‐Tcheugui et al. 2018).

Obesity has also been associated with anxiety. A recent 3‐year longitudinal study reported that individuals with obesity and no baseline mental health diagnosis had an 86% higher risk of developing a clinical anxiety disorder compared to their normal‐weight peers (de Wit et al. 2022). A recent meta‐analysis also highlighted that the frequency of anxiety in obesity was 30% higher than in normal weight (Amiri and Behnezhad 2019). The mechanisms by which obesity is associated with anxiety are both biological and psychological. The biological mechanisms implicated in obesity‐induced anxiety are four‐fold, all linked to neuroinflammatory and/or immunometabolic responses driven by an unhealthy diet and excess body weight: (i) microglial and astrocytic reactivity leading to neuroplastic changes, (ii) insulin and leptin resistance in neural cells, (iii) alterations in the kynurenine/serotonin pathway and associated glial dysfunction, and (iv) impaired hippocampal neurogenesis (Fulton et al. 2022). On the other hand, weight stigma and discrimination may contribute to negative mood states, such as body image dissatisfaction, low self‐esteem, anxiety, and depression (de Wit et al. 2022).

While research on depression‐related executive dysfunction is extensive, research on anxiety‐related executive dysfunction is comparatively less developed. Nevertheless, emerging evidence links anxiety to EF deficits (Shields et al. 2016; Warren et al. 2021). Neural correlates of anxiety involve brain regions integral to EF, including the inferior frontal gyrus, dorsolateral prefrontal cortex, and dorsal anterior cingulate cortex (Sharp et al. 2015).

Despite emerging evidence of mechanisms linking obesity to impaired EF—such as the mediating role of inflammation, and correlational findings involving obesity, cortisol, anxiety, and EF—no prior study, to our knowledge, has examined the mediating roles of inflammatory biomarkers, anxiety, and cortisol in the relationship between BMI and core EF.

Moreover, a meta‐analysis that included studies on children, adolescents, and adults concluded that obesity was associated with broad executive dysfunction, including impairments in working memory, inhibition, and cognitive flexibility (Yang et al. 2018). Another literature review (Mamrot and Hanć 2019) that focused solely on children and adolescents provided less consistent results. Specifically, only 25% of the studies assessing working memory reported significant differences between BMI groups. This increased to 70.37% for inhibitory control, and all studies assessing cognitive flexibility reported significant differences. While the overall trend suggests that higher BMI is linked to poorer executive functioning, results appear to be less consistent when considering only studies involving children and adolescents.

Given this, in the present cross‐sectional study, we use a moderated (i.e., age group: adolescent or adult) multiple mediation approach to evaluate whether TNFα, fibrinogen, cortisol, and anxiety significantly mediate the association between BMI z‐score and EF (i.e., working memory, inhibition, cognitive flexibility, and a latent component of EF) in both adolescents and adults. We hypothesise that TNFα, fibrinogen, cortisol, and anxiety will mediate the relationship between BMI z‐score and poorer EF.

## 2 Methods

### 2.1 Procedure

Participants were randomly contacted by phone, and those who expressed interest were briefly interviewed regarding general health aspects. On the first day, potential candidates were scheduled for a comprehensive medical evaluation, which included anthropometric and blood pressure measurements, further investigation of past or current medical conditions considered exclusion criteria, and a fasting blood draw carried out between 8:00 and 8:30 a.m. In the following days, individuals without any medical comorbidities underwent a neuropsychological assessment.

### 2.2 Participants

Two hundred and eighty‐nine potential candidates were recruited. The inclusion criterion was being aged between 10 and 50 years old. Exclusion criteria were having (i) underweight, (ii) psychiatric, neurological, developmental, cardiometabolic or systemic diagnosis, (iii) global cognitive impairment determined by a scalar score < 7 in the Weschler Adults Intelligence Scale‐III/Weschler Intelligence Scale for Children‐IV (WAIS‐III/WISC‐IV) vocabulary subtest, and (iv) bulimia‐like behaviours assessed by the Mini International Neuropsychiatric Interview for Bulimia or a score ≥ 20 on the Bulimia Inventory Test of Edinburgh. Bulimia was not assessed using the same questionnaire for all participants due to a change in the protocol. After applying exclusion criteria (*n* = 3 underweight, *n* = 1 binge eating, *n* = 17 metabolic syndrome) the final sample size of this cross‐sectional study was 268. Data collection took place between 2010 and 2022 and involved three independent projects funded by the Spanish government (2010–2014, 2015–2017, and 2019–2022), which recruited participants with normal weight, overweight, and obesity across various age ranges. Although we did not perform an a priori power analysis due to the retrospective nature of our analysis, our study utilises the maximum data available from these cohorts.

This study was approved by an Institutional Ethics Committee. The research was conducted in accordance with the Helsinki Declaration. Written informed consent was obtained from all participants, or their legal guardian in underage participants, prior to entry in the study. Study method and results are reported following the Strengthening the Reporting of Observational Studies in Epidemiology (STROBE) Statement for cross‐sectional studies (Von Elm et al. 2007).

### 2.3 Demographic Variables

Age, sex, self‐reported ethnicity, and socioeconomic status (SES) assessed by monthly family income were used to demographically describe both adolescent and adult samples. SES was not included as a control variable in the main analysis due to a large proportion of missing values.

### 2.4 Anthropometric Measurements

Participants were in light clothing without shoes when a trained nurse measured their height (Holtan Limited Harpenden Stadiometer) and weight (Seca 704s). BMI was calculated as kilograms/metres^2^ (kg/m^2^). The definition of overweight and obesity, when assessed using a continuous measure such as BMI, differs in interpretation between adolescents and adults. Since our methodological approach included a sample with both age groups, we transformed BMI into BMI z‐score using the R package *cdcanthro*. Although BMI‐z score can only be calculated for participants up to 20 years of age, we applied the age‐20 BMI z‐score reference for participants aged 20 and older to ensure a consistent metric across development (Must and Anderson 2006). For descriptive purposes, we classified participants as normal weight or overweight/obesity using the BMI cut‐offs from Cole and Lobstein (Cole and Lobstein 2012) for underage participants, and the 25 kg/m2 BMI cut‐off from the World Health Organization (World Health Organization 2024) for participants aged ≥ 18 years.

### 2.5 Mediators

Fibrinogen, TNFα, cortisol, and the anxiety subscale of the Hospital Anxiety and Depression Scale (HADS) were used as mediators. Concentrations of fibrinogen (*n* = 3 missing values) and cortisol (*n* = 2 missing values) were determined through PT‐derived fibrinogen assay and electrochemiluminescence (Elecsys Cortisol II), respectively. Fibrinogen values (g/L) of 139 participants were quantified with STA‐Neoplastin‐Plus reactive (STA‐Rack) (mean = 3.27, SD = 0.56, range = 2.14–5.19), while for the rest of the sample Recombiplastin 2 G reactive (ACLTOP 700) was used (mean = 3.84, SD = 0.68, range = 2.49–6.21). The inter‐assay coefficients for STA‐Rack and ACLTOP 700 were 6.52% and 3.18%, respectively, and the intra‐assay coefficient of variability for ACLTOP 700 was 0.75%. Data of the intra‐assay coefficient of STA‐Rack was not available. TNFα (*n* = 40 missing values, of which *n* = 1 was an extreme outlier that was removed from the variable) was analysed using Quantikine HS ELISA Human TNFα (R&D Systems, Ref. HSTA00E, Lot P174731 and P311121). The inter‐assay and intra‐assay coefficients of variability were 6.5% and 2%, respectively. The detection threshold was 0.156 pg/mL.

### 2.6 Neuropsychological Assessment

The neuropsychological assessment included the evaluation of core EF. Working memory was assessed using the Letter‐Number Sequencing scalar score of the WAIS‐III/WISC‐IV (Wechsler 2002, 2007), which was then converted into z‐score. Higher values represented better performance in working memory. Inhibition was evaluated using a composite score of the interference score of the Stroop Colour and Word Test (Golden 1995) and the commission errors of the Conners' Continuous Performance Test‐II (CPT‐II) (Conners 2004). The inhibition composite was calculated as follows: ((z‐score Stroop + reversed z‐score CPT‐II)/2), where higher values were indicative of better inhibitory control. Cognitive flexibility was assessed using a composite of part B minus part A of the Trail Making Test (TMT) (Reitan 1958) and the perseverative errors of the Wisconsin Card Sorting Test (WCST) (Heaton 1999). The cognitive flexibility composite was calculated as follows: (reversed z‐score TMT + reversed z‐score (WCST)/2) where higher values reflected better cognitive flexibility. A prorated composite was calculated when participants had a missing value in these cognitive variables (*n* = 2 CPT‐II and *n* = 3 WCST). All these cognitive tests have been previously administered in participants with overweight and obesity, as reported in a systematic review (Favieri et al. 2019).

### 2.7 Psychometric Properties

Reliability coefficients for the following neuropsychological tests were extracted from their respective manuals: WAIS‐III and WISC‐IV Letter‐Number Sequencing, 0.83 and 0.84 respectively (Wechsler 2002, 2007); Stroop Interference, 0.83 (Golden 1995); CPT‐II commission errors, 0.83 (Conners 2004). A recent study reported a 0.93 reliability for the WCST Perseverative Errors (Steinke et al. 2021). Concerning the TMT, as reported in Lezak (Lezak et al. 2012), reliability coefficients vary considerably depending on sample characteristics (e.g., neuropsychological condition) and differ between parts A and B.

Regarding validity, both the test manuals (Conners 2004; Golden 1995; Wechsler 2002, 2007) and various studies (Lezak et al. 2012; Sánchez‐Cubillo et al. 2009) report that these tests demonstrate adequate correlations with other established measures of the same cognitive constructs. However, factors such as age, sex, and educational level, among others, have also been identified as influential variables affecting test performance.

### 2.8 Missing Values

Some variables presented a large proportion of missing values, namely SES (*n* = 97), and TNFα (*n* = 40). As mentioned above, the data included in this paper were derived from three different protocols. Both SES and TNFα were initially included in the second protocol. TNFα levels from some participants in the first protocol were retrospectively analysed, but most participants had no remaining blood samples.

### 2.9 Statistical Analyses

Data analysis and statistical procedures were performed in R (v.4.3.2) and RStudio (v.2023.09.1). While BMI z‐score was used as a continuous variable in the main analyses, BMI group was used for descriptive purposes. Independent sample *T*‐tests, Wilcoxon tests, and Chi‐square tests were conducted to assess univariate differences between BMI groups. Normality was evaluated by Shapiro‐Wilk test. Effect sizes were calculated using R packages *lsr* and *confintr* for numerical and categorial variables, respectively.

Moderated multiple mediation analyses (*lavaan* (v.0.6.16) R package) were conducted using multigroup SEM, comparing models with a/b paths constrained to equality between adolescents and adults against unconstrained models allowing these pathways to differ across groups. Model fit comparisons were assessed using Chi‐square difference tests. We tested whether the associations between BMI z‐score (*X*) and inhibition (*Y*_*1*_), cognitive flexibility (*Y*_*2*_), working memory (*Y*_*3*_), and latent EF construct (*Y*_*4*_) were mediated by cortisol (*M*_*1*_), TNFα (*M*_*2*_), fibrinogen (*M*_*3*_) and HADS anxiety (*M*_*4*_) in adolescents and adults (*X → M*_*1*_ */M*_*2*_ */M*_*3*_ */M*_*4*_ *→ Y*_*n*_). The latent EF construct, composed of working memory, inhibition, and cognitive flexibility, was estimated via confirmatory factor analysis (CFA) within a multigroup SEM framework. All models were adjusted for age, sex, and estimated intelligence, as these variables may influence EF performance (Lezak et al. 2012). If a participant presented a missing value in any of the Y, X, M or adjusted variables, the individual was removed from the mediation analysis (listwise deletion procedure). We considered a mediator significant if the 95% confidence interval (CI) from its indirect effect did not include zero. The 95% CI was calculated by bootstrapping 1000 simulations. Multiple linear assumptions of normality and homoscedasticity were checked using the functions in the *olsrr* (v.0.5.3) R package.

Sensitivity analyses were performed excluding participants with high‐sensitivity C‐reactive protein levels > 10 mg/L (Supporting Information S1: Table S1). Additionally, moderated simple mediation analyses were conducted for mediators that were significant in the moderated multiple mediation analysis (Supporting Information S1: Table S2).

## 3 Results

### 3.1 Descriptive Characteristics

Descriptive characteristics of both adolescent and adult samples are presented in Tables 1 and 2, respectively. Overall, individuals with overweight/obesity had higher levels of fibrinogen and TNFα. Cortisol levels were lower in this group, although the difference reached significance only in the adult sample. Lower inhibitory control was also observed among those with overweight/obesity.

**TABLE 1:** Descriptive characteristics of the adolescent sample.

	NW (*n* = 54)		OW/OB (*n* = 75)		Test statistic	*p* value	Effect size
	Mean (SD)	Range	Mean (SD)	Range			
Demographic variables							
Age (years)	15 (2.1)	11, 19	14.7 (1.9)	11, 19	*W* = 2178	0.46	*d* = 0.16
Sex (*n* females (%))	30 (55.5)	—	33 (44)	—	Chi^2^ = 1.24	0.26	*V* = 0.11
Self‐reported ethnicity							
Latinx (*n* (%))	1 (1.8)	—	5 (6.6)	—	Chi^2^ = 0.79	0.37	*V* = 0.11
Spaniard (*n* (%))	53 (98.2)		68 (90.6)				
NA (*n* (%))	0		2 (2.8)				
Monthly family income (€)							
300–899 (*n* (%))	1 (1.8)	—	1 (1.3)	—	Chi^2^ = 0.81	0.93	*V* = 0.15
900–1499 (*n* (%))	4 (7.4)		4 (5.3)				
1500–2099 (*n* (%))	7 (12.9)		8 (10.6)				
2100–2699 (*n* (%))	3 (5.5)		3 (4)				
≥ 2700 (*n* (%))	1 (1.8)		3 (4)				
NA (*n* (%))	38 (70.4)		56 (74.6)				
Anthropometric measure							
BMI z‐score	−0.23 (0.62)	−1.56, 0.81	1.89 (0.37)	1, 3.01	*W* = 0	< 0.001	*d* = 4.27
Mediators							
TNFα (pg/mL)	0.7 (0.33)	0.25, 1.95	0.9 (0.40)	0.26, 2.52	*W* = 897	0.002	*d* = 0.54
NA (*n*)	8		16				
Fibrinogen (g/L)	3.2 (0.59)	2.23, 5.47	3.83 (0.71)	2.56, 6.21	*W* = 992	< 0.001	*d* = 0.86
NA (*n*)	1		0				
Cortisol (nmol/L)	395.85 (164.5)	146.3, 862.8	363.56 (142)	121, 777	*W* = 2178	0.31	*d* = 0.21
HADS anxiety	5.24 (2.7)	0, 11	4.84 (3.18)	0, 14	*W* = 2220	0.34	*d* = 0.13
Cognitive functions							
Estimated intelligence	11.2 (2.57)	7, 19	10.4 (2.3)	7, 17	*W* = 2329	0.14	*d* = 0.30
Inhibition (composite z‐score)	−0.14 (0.62)	−17, 2.16	−0.43 (0.98)	−1.74, 0.85	*T* = 2.53	0.01	*d* = 0.44
Cognitive flexibility (composite z‐score)	−0.04 (0.73)	−2.6, 0.93	−0.2 (0.76)	−2.48, 0.97	*W* = 2275	0.23	*d* = 0.21
Working memory (z‐score)	0.02 (1.1)	−2.6, 2.25	−0.19 (1.12)	−2.65, 3	*W* = 2240	0.30	*d* = 0.19
Latent EF component	−0.55 (0.22)	−1.07, −0.09	−0.35 (0.20)	−0.78, 0.05	*W* = 674	< 0.001	*d* = 0.94

*Note:* We provide mean (SD) and range for numerical variables, and *n* counts (%) for categorical variables. Effect size: Cohen's *d* is reported for numerical variables and Cramer's *V* for categorical variables. Estimated intelligence was assessed by the Weschler Adults Intelligence Scale‐III/Weschler Intelligence Scale for Children‐IV (WAIS‐III/WISC‐IV) Vocabulary subtest scalar score. Inhibition was evaluated using a composite score of the interference score of the Stroop Colour and Word Test and the commission errors of the Conners' Continuous Performance Test‐II (CPT‐II). Cognitive flexibility was assessed using a composite score of part B minus part A of the Trail Making Test and the perseverative errors of the Wisconsin Card Sorting Test (WCST). Working memory was assessed using the Letter‐Number Sequencing scalar score of the WAIS‐III/WISC‐IV. The latent EF component was estimated by deriving individual factor scores from the moderated multiple mediation model using the lavPredict() function implemented in the lavaan R package.

Abbreviations: BMI, body mass index; HADS, hospital anxiety and depression scale; NA, not available; NW, normal weight; OW/OB, overweight/obesity.

**TABLE 2:** Descriptive characteristics of the adult sample.

	NW (*n* = 58)		OW/OB (*n* = 80)		Test statistic	*p* value	Effect size
	Mean (SD)	Range	Mean (SD)	Range			
Demographic variables							
Age (years)	29.3 (6.8)	20, 42	33.5 (7.8)	20, 49	*W* = 1616	0.002	*d* = 0.56
Sex (*n* females (%))	37 (63.7)	—	48 (60)	—	Chi^2^ = 0.07	0.78	*V* = 0.03
Self‐reported ethnicity							
Latinx (*n* (%))	6 (10.3)	—	5 (6.2)	—	Chi^2^ = 0.26	0.60	*V* = 0.07
Spaniard (*n* (%))	52 (89.7)		73 (91.3)				
NA (*n* (%))	0		2 (2.5)				
Monthly family income (€)							
300–899 (*n* (%))	2 (3.45)	—	4 (5)	—	Chi^2^ = 1.43	0.83	*V* = 0.10
900–1499 (*n* (%))	9 (15.52)		15 (18.75)				
1500–2099 (*n* (%))	20 (34.48)		28 (35)				
2100–2699 (*n* (%))	10 (17.24)		17 (21.25)				
≥ 2700 (*n* (%))	15 (25.86)		15 (18.75)				
NA (*n* (%))	2 (3.45)		1 (1.25)				
Anthropometric measure							
BMI z‐score	−0.02 (0.55)	−1.4, 0.76	1.58 (0.56)	0.64, 3.37	*W* = 20	< 0.001	*d* = 2.86
Mediators							
TNFα (pg/mL)	0.7 (0.26)	0.2, 1.79	0.9 (0.26)	0.32, 1.57	*W* = 943	< 0.001	*d* = 0.73
NA (*n*)	7		9				
Fibrinogen (g/L)	3.2 (0.65)	2.14, 5	3.91 (0.68)	2.71, 5.83	*W* = 970	< 0.001	*d* = 1.05
NA (*n*)	1		1				
Cortisol (nmol/L)	589.88 (201.4)	222.2, 1315	459.83 (149.2)	178.9, 917.8	*W* = 3260	< 0.001	*d* = 0.75
HADS anxiety	4.24 (2.8)	0, 11	4.07 (2.69)	0, 11	*W* = 2408	0.70	*d* = 0.06
Cognitive functions							
Estimated intelligence	11.53 (1.96)	7, 16	11.84 (1.96)	8, 17	*W* = 2152	0.46	*d* = 0.15
Inhibition (composite z‐score)	0.43 (0.57)	−1.31, 1.87	0.18 (0.64)	−1.44, 1.55	*T* = 2.37	0.02	*d* = 0.40
Cognitive flexibility (composite z‐score)	0.19 (0.58)	−1.08, 1.27	0.07 (0.83)	−2.9, 1.29	*W* = 2405	0.71	*d* = 0.15
Working memory (z‐score)	0.18 (0.78)	−1.42, 3.07	0.03 (0.92)	−1.83, 2.25	*W* = 2516	0.39	*d* = 0.17
Latent EF component	1.08 (0.36)	0.39, 2.24	0.99 (0.42)	−0.24, 1.93	*W* = 1930	0.41	*d* = 0.24

*Note:* We provide mean (SD) and range for numerical variables, and *n* counts (%) for categorical variables. Effect size: Cohen's *d* is reported for numerical variables and Cramer's *V* for categorical variables. Estimated intelligence was assessed by the Weschler Adults Intelligence Scale‐III/Weschler Intelligence Scale for Children‐IV (WAIS‐III/WISC‐IV) Vocabulary subtest scalar score. Inhibition was evaluated using a composite score of the interference score of the Stroop Colour and Word Test and the commission errors of the Conners' Continuous Performance Test‐II (CPT‐II). Cognitive flexibility was assessed using a composite score of the part B minus part A of the Trail Making Test and the perseverative errors of the Wisconsin Card Sorting Test. Working memory was assessed using the Letter‐Number Sequencing scalar score of the WAIS‐III/WISC‐IV. The latent EF component was estimated by deriving individual factor scores from the moderated multiple mediation model using the lavPredict() function implemented in the lavaan R package.

Abbreviations: BMI, body mass index; HADS, hospital anxiety and depression scale; NA, not available; NW, normal weight; OW/OB, overweight/obesity.

### 3.2 Moderated Multiple Mediation Analyses

Moderated multiple mediation analyses were conducted using multigroup SEM, comparing constrained and unconstrained models. Chi‐square difference tests of model fit suggested using the unconstrained model for both working memory (Δχ^2^ (9) = 19.1, *p* = 0.02) and the latent EF construct (Δχ^2^ (9) = 16.3, *p* = 0.06). Although the latter was marginally non‐significant, model fit favoured the unconstrained solution, as indicated by a lower Akaike Information Criterion (AIC) value (5038.8 vs. 5089.8). For cognitive flexibility and inhibition, Chi‐square difference tests suggested using constrained models (Δχ^2^ (9) = 16.3, *p* = 0.06 and Δχ^2^ (9) = 12.7, *p* = 0.17, respectively).

#### 3.2.1 Unconstrained Models (Different Paths Between Adolescents and Adults): Working Memory and the Latent EF Construct

**FIGURE 1:**
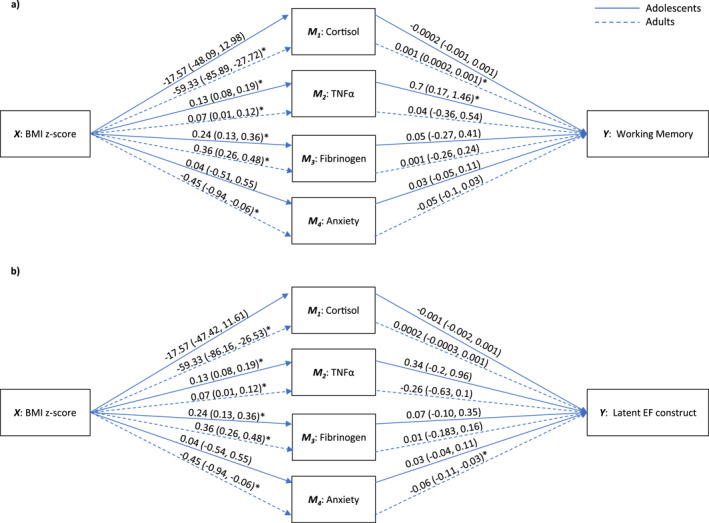
Moderated multiple mediation analysis between BMI z‐score and (a) working memory and (b) the latent EF construct. These models followed different pathways for adolescents and adults (unconstrained models). Parallel mediators are cortisol, TNFα, fibrinogen, and anxiety. Abbreviations: BMI, body mass index, EF, executive functions, TNFα, tumour necrosis factor alpha.

Our results suggested that in adolescents, TNFα significantly mediated the relationship between BMI z‐score and working memory (beta indirect effect = 0.09, 95% CI [0.03, 0.19]). In adults, cortisol significantly mediated the relationship between BMI z‐score and working memory (beta indirect effect = −0.06, 95% CI [−0.13, −0.01]). Also, anxiety significantly mediated the relationship between BMI z‐score, working memory, and the latent EF construct (beta indirect effect = 0.02, 95% CI [0.0001, 0.07] and beta indirect effect = 0.03, 95% CI [ 0.004, 0.08], respectively). We were not able to reject the null hypothesis for the rest of mediators. Figure 1 and Table 3 provide the estimates for the *a* path, *b* path, direct effect, and indirect effect. Sensitivity analysis excluding participants with high‐sensitivity C‐reactive protein levels > 10 mg/L yielded consistent results (Supporting Information S1: Table S1).

**TABLE 3:** Moderated multiple mediation estimates with four parallel mediators for the unconstrained models.

Outcome (Y)	Age group	Direct and indirect effects		Estimates with 95% CI
Working memory	Adolescents	Direct effect		−0.17 (−0.38, 0.04)
		Indirect effects	TNFα	0.09 (0.03, 0.19)^a^
			Fibrinogen	0.01 (−0.07, 0.1)
			Cortisol	0.005 (−0.02, 0.05)
			Anxiety	0.001 (−0.02, 0.04)
	Adults	Direct effect		−0.04 (−0.19, 0.14)
		Indirect effects	TNFα	0.003 (−0.02, 0.05)
			Fibrinogen	0.001 (−0.09, 0.09)
			Cortisol	−0.06 (−0.13, −0.01)^a^
			Anxiety	0.02 (0.0001, 0.07)^a^
Latent EF construct	Adolescents	Direct effect		0.00001 (−0.23, 0.16)
		Indirect effects	TNFα	0.04 (−0.03, 0.12)
			Fibrinogen	0.02 (−0.02, 0.01)
			Cortisol	0.01 (−0.01, 0.08)
			Anxiety	0.001 (−0.02, 0.03)
	Adults	Direct effect		−0.08 (−0.22, 0.03)
		Indirect effects	TNFα	−0.02 (−0.05, 0.01)
			Fibrinogen	0.003 (−0.07, 0.05)
			Cortisol	−0.02 (−0.09, 0.02)
			Anxiety	0.03 (0.005, 0.09)^a^

*Note:* Variables of the moderated multiple mediation models included body mass index z‐score (X), cortisol (M1), TNFα (M2), fibrinogen (M3) anxiety (M4), working memory (Y1), latent EF construct (Y2), and age group (moderator). Sex, age, and estimated intelligence were used as covariates.

Abbreviation: EF, executive function; TNFα, tumour necrosis factor alpha.

^a^ Significant 95% confidence interval.

#### 3.2.2 Constrained Models (Equal Paths Between Adolescents and Adults): Inhibition and Cognitive Flexibility

**FIGURE 2:**
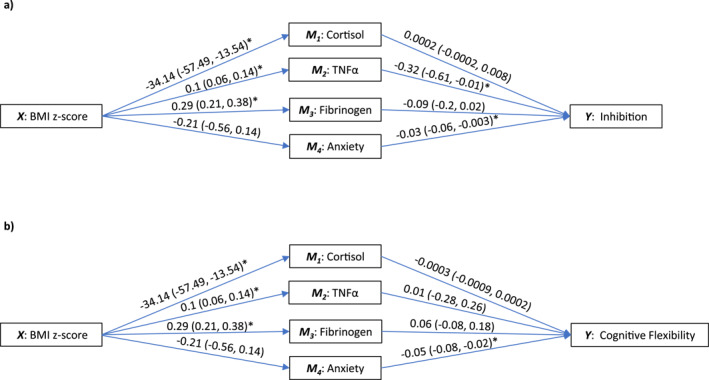
Moderated multiple mediation analysis between BMI z‐score and (a) inhibition and (b) cognitive Flexibility. These models followed equal pathways for adolescents and adults (constrained models). Parallel mediators are cortisol, TNFα, fibrinogen, and anxiety. Abbreviations: BMI, body mass index, TNFα, tumour necrosis factor alpha.

In the full sample, TNFα significantly mediated the relationship between BMI z‐score and inhibition (beta indirect effect = −0.03, 95% CI [−0.06, −0.002]). We were not able to reject the null hypothesis for the rest of mediators. Figure 2 and Table 4 provide the estimates for the *a* path, *b* path, direct effect, and indirect effect. Sensitivity analysis excluding participants with high‐sensitivity C‐reactive protein levels > 10 mg/L yielded consistent results for inhibition. In the cognitive flexibility model, the Chi‐square difference test of model fit indicated that an unconstrained model should be used. Consequently, different pathways for adults and adolescents were explored, and anxiety reached statistical significance in adults (beta indirect effect = 0.04, 95% CI [0.007, 0.11]) (Supporting Information S1: Table S1).

**TABLE 4:** Moderated multiple mediation estimates with four parallel mediators for the constrained models.

Outcome (Y)	Direct and indirect effects		Estimates with 95% CI
Inhibition	Direct effect		−0.03 (−0.12, 0.06)
	Indirect effects	TNFα	−0.03 (−0.06, −0.002)^a^
		Fibrinogen	−0.03 (−0.06, 0.01)
		Cortisol	−0.01 (−0.03, 0.004)
		Anxiety	0.01 (−0.002, 0.02)
Cognitive flexibility	Direct effect		−0.11 (−0.21, −0.02)^a^
	Indirect effects	TNFα	0.001 (−0.03, 0.03)
		Fibrinogen	0.02 (−0.02, 0.06)
		Cortisol	0.01 (−0.003, 0.04)
		Anxiety	0.01 (−0.01, 0.04)

*Note:* Variables of the moderated multiple mediation models included body mass index z‐score (X), cortisol (M1), TNFα (M2), fibrinogen (M3) anxiety (M4), inhibition (Y1), cognitive flexibility (Y2), and age group (moderator). Sex, age, and estimated intelligence were used as covariates.

Abbreviation: TNFα, tumour necrosis factor alpha.

^a^ Significant 95% confidence interval.

## 4 Discussion

In this study, evaluated whether TNFα, fibrinogen, cortisol, and anxiety mediate the association between BMI z‐score and EF performance—specifically working memory, inhibition, cognitive flexibility, and a latent EF component—in adolescents and adults, using a moderated multiple mediation approach. We examined the mediating roles of these biological and psychological variables and found that some pathways varied between adolescents and adults, while others did not. Specifically, TNFα mediated the association between BMI z‐score and working memory only in adolescents, whereas in adults, cortisol and anxiety mediated this association. Additionally, in adults, anxiety also mediated the relationship between BMI z‐score and the latent EF construct. In the full sample, TNFα significantly mediated the relationship between BMI z‐score and inhibition.

### 4.1 Inflammation as Mediator

Our results suggested that TNFα significantly mediated the relationship between BMI z‐score and working memory in adolescents, as well as between BMI z‐score and inhibition in the full sample. In adults, the indirect effect was negative. In adolescents, and contrary to our expectations, it was positive. As for fibrinogen, we were unable to reject the null hypothesis, and thus could not confirm its significance.

A recent review (Mekhora et al. 2024) examined the association between inflammation and cognitive function in adults. First, they summarised nine randomized controlled trials in which acute inflammatory responses were induced using endotoxins, and cognitive performance was assessed before and after stimulation. Four of these studies did not report significant effects. Second, they reviewed 19 meta‐analyses investigating the relationship between inflammation and cognitive decline or cognitive‐related conditions, of which two reported no significant findings. Overall, the studies suggested that elevated levels of pro‐inflammatory cytokines were associated with greater cognitive dysfunction, aligning with our results in the full sample. However, the review also highlighted the presence of null findings, which the authors attributed to variations in study methodology or sample characteristics.

While methodological considerations could partly explain the observed significant positive indirect effect in adolescents, an alternative hypothesis should be considered. Although TNFα emerged as a significant mediator, it is likely one of several biological intermediaries influencing the direction and magnitude of the effect. Pro‐inflammatory states are typically accompanied by compensatory anti‐inflammatory responses, including the release of interleukins such as IL‐1Ra, IL‐4, IL‐5, IL‐10, and IL‐11 (Al‐Mansoori et al. 2022). In a mouse model, depressive‐like behaviours induced via a learnt helplessness paradigm, known to promote pro‐inflammatory cytokine production, led to deficits in novel object recognition and spatial working memory. Notably, these deficits were reversed following administration of the anti‐inflammatory cytokine IL‐10 (Worthen et al. 2020). We suggest that the dynamic interplay between pro‐ and anti‐inflammatory cytokines, alongside other biological intermediates, may have differential effects on cognitive outcomes across developmental stages.

### 4.2 Cortisol as Mediator

Our results suggested that cortisol significantly mediated the relationship between a higher BMI z‐score and a poorer working memory in adults. This mediating effect of cortisol on working memory aligns with the results of a meta‐analysis that described the association between cortisol administration and short‐term impairments in working memory—an effect that reversed over time (Shields et al. 2015). Downstream responses triggered by cortisol receptor activation can be either nongenomic (e.g., mediated via membrane‐bound corticosteroid receptors or direct protein‐protein interactions, often with a rapid onset within seconds to minutes) or genomic (e.g., modulated by gene expression, typically with a relatively slow onset over hours to days) (Haque et al. 2020). Thus, our cross‐sectional findings may differ qualitatively from effects observed in longitudinal designs.

In the latent EF component model, we were not able to reject the null hypothesis for cortisol; however, anxiety raised as a significant mediator. The effects of cortisol on executive functions may differ across specific domains. In the previously mentioned meta‐analysis (Shields et al. 2015), cortisol administration was associated with short‐term impairments in working memory and with enhanced inhibition—which reversed over time—and non‐significant associations with cognitive flexibility. Thus, creating a latent EF component may have blurred the cortisol effects seen in working memory.

Interestingly, we observed a significant mediating role of cortisol only in adults. Activation of the HPA axis and the release of glucocorticoids have the greatest impact on brain structures that are still developing at the time of exposure or on regions that are particularly vulnerable (e.g., areas most affected by ageing). The adolescent brain is characterised by a still developing prefrontal cortex, which shows higher levels of GR mRNA expression than in infancy, young adulthood, or old age. Excessive exposure to glucocorticoids during this sensitive period of synaptic organisation may alter the developmental trajectory of neuronal connections, leading to an incubation period in which the effects of glucocorticoids do not manifest immediately, but emerge later when synaptic organisation is complete (Lupien et al. 2009). While this may help explain why significant effects were not observed in adolescents, future longitudinal studies should explore this potential age‐dependent effects of cortisol on executive functions.

### 4.3 Anxiety as Mediator

In our study, anxiety mediated the relationship between BMI z‐score and working memory, as well as between BMI z‐score and the latent EF component, but only in adults. Anxiety is a heterogeneous construct, comprising both anxious apprehension (trait anxiety) and anxious arousal (state anxiety). A recent study that used this classification found that anxious arousal was associated with broad executive dysfunction, whereas anxious apprehension was linked specifically to deficits in cognitive flexibility (Warren et al. 2021). In our study, we were unable to distinguish between these dimensions of anxiety, which would have provided greater insight into the interpretation of our findings. Nonetheless, we had hypothesised a negative mediation effect of anxiety on the relationship between BMI z‐score and EF, but found the opposite.

In a study with limited sample size, trait anxiety was associated with deficits in executive control, whereas state anxiety was linked to overactive alerting and orienting networks (Pacheco‐Unguetti et al. 2010). Applying these findings to our results, it is possible that heightened alerting attention may have helped participants focus, thereby enhancing their performance on EF tasks (Ursache and Raver 2014). Consistent with this interpretation, another small study reported that state anxiety was associated with improved inhibition and cognitive flexibility (Kofman et al. 2006). While these studies offer a possible explanation for our unexpected findings, they are limited by their small sample sizes and cross‐sectional designs, and thus should be interpreted with caution.

### 4.4 Limitations

An important limitation of the present study concerns the reliability and sensitivity of the neuropsychological tasks selected to detect individual differences in a healthy sample. Although all these tasks have been previously used in cognitively normative populations, they are mostly administered for clinical purposes. Even in neurological patients, inconsistencies in task performance are often attributed to variability in pathology severity or differences in the anatomical location of brain damage (Lezak et al. 2012; Mitrushina et al. 2005). In healthy samples such as ours, the low between‐subject variance may make these tasks insufficiently sensitive and reliable for detecting subtle cognitive differences (Hedge et al. 2018). As a result, it is possible that true associations were not detected in our data due to the measurement limitations of the tasks used.

Cortisol measurement is another limitation. Circulating cortisol measures both free (i.e., biologically active) and bound cortisol, which may lead to potential inaccuracies in reflecting the biologically active cortisol. Additionally, the differences in cortisol observed between BMI groups may be time‐specific (Luecken and Gallo 2008). Because we relied on a single morning cortisol sample, we were unable to evaluate potential differences in circadian cortisol patterns across groups.

Moreover, our cross‐sectional design provides only preliminary support for the proposed causal model and does not establish the temporal sequence required to show that changes in the mediator preceded changes in the outcome variable. Finally, information regarding years of evolution of overweight/obesity was only available for 39.7% of the participants with this BMI category and, therefore, could not be included as a confounding factor in analysis.

Future research employing a longitudinal design, incorporating neuropsychological tasks with greater ecological validity and multiple cortisol samples, and accounting for potential confounders such as socioeconomic status and duration of obesity, will help clarify the mediating mechanisms underlying the long‐term impact of adiposity on EF.

## 5 Conclusions

In this study, we aimed to evaluate whether TNFα, fibrinogen, cortisol, and anxiety mediate the relationship between BMI z‐score and EF in a sample of adolescents and adults ranging in BMI from normal‐weight to obesity. Using a moderated multiple mediation model, we found preliminary evidence that TNFα, cortisol, and anxiety, but not fibrinogen, mediate this relationship. While the significance and direction of the indirect effects varied between adolescents and adults in some models, our results suggest that inflammation, cortisol, and anxiety are biologically and psychologically plausible mechanisms through which BMI can influence cognitive performance.

## Ethics Statement

All procedures performed involving human participants were in accordance with the ethical standards of the institutional and/or national research committee and with the 1964 Helsinki declaration and its later amendments or comparable ethical standards. This study was approved by the Institutional Ethics Committee of the University of Barcelona (Institutional Review Board IRB00003099, assurance number FWA00004225).

## Consent

Written informed consent was obtained from all participants, or their legal guardian in underage participants, prior to entry in the study.

## Conflicts of Interest

The authors declare no conflicts of interest.

## Supporting information

Supporting Information S1

## Data Availability

The data that support the findings of this study are not publicly available due to their containing information that could compromise the privacy of research participants but are available from the corresponding author (MAJ) upon reasonable request.
