# Supplementary material for: Cortisol, Anxiety, and TNFα Mediate the Relationship Between BMI and Executive Functions
Source: Stress Health. 2025 Jul 12;41(4):e70077. doi: 10.1002/smi.70077 (PMC12255384; doi:10.1002/smi.70077)
Supplement: Supplementary file 1 — Supporting Information S1 [file SMI-41-e70077-s001.docx]

**Table S1. Moderated multiple mediation estimates without participants with high-sensitivity C-reactive protein levels >10 mg/L (n = 253) in constrained (i.e., inhibition) and unconstrained (i.e., cognitive flexibility, working memory, and the latent EF construct) models.**

| **Outcome (Y)** | **Age group** | **Direct and indirect effects** | | | **Estimates with 95% CI** |
| --- | --- | --- | --- | --- | --- |
| **Inhibition** | **Full sample** | Direct effect | | | -0.02 (-0.119, 0.084) |
|  |  | Indirect effect | *TNFα* | | -0.03 (-0.07, -0.002)* |
|  |  | *Fibrinogen* | | | -0.03 (-0.07, 0.01) |
|  |  | *Cortisol* | | | -0.01 (-0.04, 0.01) |
|  |  | *Anxiety* | | | 0.01 (-0.003, 0.02) |
| **Cognitive flexibility** | **Adolescents** | Direct effect | | | -0.11 (-0.26, 0.03) |
|  |  | Indirect effect | *TNFα* | | 0.04 (-0.003, 0.11) |
|  |  | *Fibrinogen* | | | -0.01 (-0.07, 0.04) |
|  |  | *Cortisol* | | | 0.02 (-0.004, 0.07) |
|  |  | *Anxiety* | | | -0.004 (-0.04, 0.02) |
|  | **Adults** | Direct effect | | | -0.18 (-0.34, -0.02)* |
|  |  | Indirect effect | *TNFα* | | -0.01 (-0.079, 0.03) |
|  |  | *Fibrinogen* | | | 0.01 (-0.075, 0.08) |
|  |  | *Cortisol* | | | 0.02 (-0.044, 0.07) |
|  |  | *Anxiety* | | | 0.04 (0.007, 0.11)* |
| **Working memory** | **Adolescents** | Direct effect | |  | -0.17 (-0.38, 0.04) |
|  |  | Indirect effects | | *TNFα* | 0.09 (0.03, 0.19)* |
|  |  |  | | *Fibrinogen* | 0.01 (-0.07, 0.1) |
|  |  |  | | *Cortisol* | 0.005 (-0.02, 0.05) |
|  |  |  | | *Anxiety* | 0.001 (-0.02, 0.04) |
|  | **Adults** | Direct effect | |  | -0.04 (-0.19, 0.14) |
|  |  | Indirect effects | | *TNFα* | 0.003 (-0.02, 0.05) |
|  |  |  | | *Fibrinogen* | 0.001 (-0.09, 0.09) |
|  |  |  | | *Cortisol* | -0.06 (-0.13, -0.01)* |
|  |  |  | | *Anxiety* | 0.02 (0.0001, 0.07)* |
| **Latent EF construct** | **Adolescents** | Direct effect | |  | 0.00001 (-0.23, 0.16) |
|  |  | Indirect effects | | *TNFα* | 0.04 (-0.03, 0.12) |
|  |  |  | | *Fibrinogen* | 0.02 (-0.02, 0.01) |
|  |  |  | | *Cortisol* | 0.01 (-0.01, 0.08) |
|  |  |  | | *Anxiety* | 0.001 (-0.02, 0.03) |
|  | **Adults** | Direct effect | |  | -0.08 (-0.22, 0.03) |
|  |  | Indirect effects | | *TNFα* | -0.02 (-0.05, 0.01) |
|  |  |  | | *Fibrinogen* | 0.003 (-0.07, 0.05) |
|  |  |  | | *Cortisol* | -0.02 (-0.09, 0.02) |
|  |  |  | | *Anxiety* | 0.03 (0.005, 0.09)* |

Models of multiple mediation were adjusted by sex, age, and estimated intelligence. Abbreviations: EF: executive functions; TNFα: tumor necrosis factor alpha. *Significant 95% confidence interval.

**Table S2. Moderated simple mediation estimates for significant mediators in the multiple mediation analysis.**

| **Outcome (Y)** | **Age group** | **Direct and indirect effects** | | **Estimates with 95% CI** |
| --- | --- | --- | --- | --- |
| **Working memory** | **Adolescents** | Direct effect |  | -0.17 (-0.38, 0.04) |
|  |  | Indirect effects | *TNFα* | 0.09 (0.03, 0.19)* |
|  | **Adults** | Direct effect |  | -0.04 (-0.19, 0.14) |
|  |  | Indirect effects | *Cortisol* | -0.06 (-0.13, -0.01)* |
|  |  |  | *Anxiety* | 0.02 (0.0001, 0.07)* |
| **Latent EF construct** | **Adults** | Direct effect |  | -0.08 (-0.22, 0.03) |
|  |  | Indirect effects | *Anxiety* | 0.03 (0.005, 0.09)* |
| **Inhibition** | **Full sample** | Direct effect |  | -0.06 (-0.14, 0.02) |
|  |  | Indirect effects | *TNFα* | -0.03 (-0.08, -0.004)* |

Models of the moderated mediation were adjusted by sex, age, and estimated intelligence. Abbreviations: EF: executive functions; TNFα: tumor necrosis factor alpha.

*Significant 95% confidence interval.
